# Myths of past biases and progress in biology

**DOI:** 10.1007/s12064-023-00403-2

**Published:** 2023-09-23

**Authors:** Jani Raerinne

**Affiliations:** University of Helsinki, Helsinki, Finland

**Keywords:** Competition, Darwin, Mutualism, Predation, Research biases, Victorian biology

## Abstract

Two ideas are popular among biologists. The first idea is concerned with the biased nature of biology, especially the idea that biologists have overemphasized the importance of competition in the past. The second idea is concerned with progress in correcting for biases, namely, that the biased nature of biology decreases with time. To test these ideas, data on the popularity of interaction topics, such as competition, predation, and mutualism, was collected from articles published in biology journals. Research biases should be visible in publication data as systematic over- and underemphases regarding the popularity of alternative, viable research topics. Were the two ideas correct, data should show that the popularity of a historically dominant topic(s) diminishes with time, whereas the popularity of historically marginal, alternative topics increases with time. The data show that the two ideas are false. According to publication data, the biased nature of biology increases with time, which is a sign of regress rather than progress in biology.

## Introduction

The view of nature as ‘red in tooth and claw’, as a jungle which reflected the industrial society in which the new sciences were being developed, strongly colored the outlook of Victorian biology. This social background… placed heavy emphasis on the inevitability of competition, a scientific view which served to justify the dog-eat-dog world of the *laissez-faire* market economy (Risch and Boucher 1976: 8–9).

[D]espite the wealth of available information, mutualism was not a prominent concept in ecology through most of the 20th century. Ecologists … focused almost exclusively on competition and predation… The first major conceptual advances only began in the 1960s, and it is only since the mid-1980s that the field of mutualism has truly flourished (Bronstein 2009: 1161).

The role of negative interactions… has been a focus of considerable research for at least decades. … However, in the last decade, a series of studies have highlighted the important role that positive interactions played in shaping the structure of communities. This renewed interest in positive interactions had led to a reconsideration of the niche concept (Rodriguez-Cabal et al. 2012: 36).

Two popular ideas among biologists go back over one hundred years (e.g. Kropotkin 1890a, b). The first is that biology was biased because biologists have overemphasized the importance of competition (and predation) in the past. The second idea is concerned with subsequent progress. While biology might have been biased in the past, biologists have rid themselves of their ancestors’ biases. Or, at least, biologists have started to correct for biases. An interesting aspect of these ideas is that each new generation rediscovers them.

This study is based on data collected from articles published in journals to test these ideas. Publication data indicate what were dominant and marginal research areas and topics of a scientific community provided that the sample of investigated journals is representative and the popularity of topics is measured by reliable indicators. Research biases should be visible in publication data as systematic over- and underemphases regarding the popularity of alternative, viable research topics. Were the old ideas correct, publication data should show that the popularity of historically dominant topics diminishes with time, whereas the popularity of historically marginal, alternative topics increases with time.

Data were collected on interaction topics. A search was conducted for hits on the seven most used and theoretically important negative (amensalism, competition, parasitism, and predation) and positive (commensalism, symbiosis, and mutualism) interaction terms, which biologists have used since the nineteenth century. These terms describe main complementary interaction types, which are needed for the taxonomy of interactions in nature.^1^ More recently coined terms are typically specifications of these general types. For instance, ‘herbivory’ is a form of predation. ‘Allelopathic’ interactions are often understood as specific cases of competition.

The relative importance of these interaction topics was and still is a central controversy in debates concerning main biological theories, such as evolutionary theory. A central issue in these debates concerns research biases. A research bias has the effect that the relative importance of a research topic departs systematically and significantly from its relative importance in nature.

In section "Materials and methods", I describe the methods used in this study. In section "Results", I describe results based on different bias measures, which provide conflicting results concerning the biased nature of biology, especially in the past. In section "Discussion", the results are discussed, the main claim being that the evidential support of the old ideas is questionable. Evidence comes from unreliable bias measures and/or controversial readings of Malthus, Darwin, and Kropotkin. If the questionable data sources are substituted with more reliable ones, the data indicate that the biased nature of biology increases, rather than decreases, with time, in sharp contrast to the old ideas.

## Materials and methods

A search for the most used negative and positive interaction terms from published articles in journals was conducted via JSTOR (https://www.jstor.org/action/showAdvancedSearch). I searched for hits for the following terms: ‘amensalism,’ ‘competition,’ ‘parasitism,’ ‘predation,’ ‘commensalism,’ ‘symbiosis,’ and ‘mutualism.’ The search covered abstracts, item titles, titles, and key words of articles. These data were recorded as hits for each term.

Positive interactions refer to commensalism (+/0), mutualism (+/+), and symbiosis (+/+)/(+/0), in which at least one participant receives positive effects from the interaction without negatively affecting the other participant. In the case of negative interactions, such as amensalism (−/0), competition (−/−), parasitism (+/−), and predation (+/−), at least one participant negatively affects the other.

I have included symbiosis among positive interactions. Many biologists make this connection [cf. Boucher et al. (1982), Bronstein (1994), Martin and Schwab (2013)].

Biologists have used these general technical terms at least since the 1870s/1880s, in contrast with more recent, less general terms (e.g. herbivory (+/−) and neutralism (0/0)) and vague or redundant terms that never become popular (e.g. cooperation (+/+), mutual aid (+/+), and struggle^2^).

Number of hits data are unsuitable as a measure of biases within a journal between its different periods and between different journals for the same or different periods, since the number of published articles varies in journals with time. I used three different measures to estimate research biases. In all cases, the number of hits data was converted into dimensionless numbers that allow one to estimate the popularity of research topics in a journal between different periods and between different journals for the same and for different periods.

The first measure is called a negative dominance (ND) value. ND values were calculated by dividing the sum of hits for all negative interaction terms (amensalism, competition, predation, and parasitism) by the sum of hits for all negative and positive interaction terms. The closer an ND value is to 100%, the higher the proportion and dominance of negative interactions. Conversely, the closer an ND value is to 0%, the higher the proportion and dominance of positive interactions. The closer an ND value is to 50%, the closer the proportion is to being equal in value, which indicates a lack of dominance and bias in research.

The second measure is called a competition dominance (CD) value. CD values were calculated by dividing the sum of hits for competition by the sum of hits for all negative and positive interaction terms. The closer a CD value is to 100%, the higher the dominance of competition. Similar bias measures can be constructed for other terms, such as predation. Since the prevailing idea is that competition dominated in the past, only CD values are discussed here. The popularity of other negative interaction terms can be determined by comparing ND values to CD values (cf. figures in section "ND and CD values"). Low CD values associated with high ND values imply the high popularity of predation and parasitism within interaction topics (but not amensalism, see below), whereas high CD values associated with high ND values imply the high popularity of competition within interaction topics.

There are two versions of the old ideas. The more popular version is concerned with past biases in *competition*. This bias should be visible in journals’ CD value data. The less popular version is concerned with the past biases in *negative interactions*, mainly competition and predation. This bias should be visible in journals’ ND value data.

The third bias measure is the percentages for individual interaction terms. The number of articles referring to search terms were converted into estimations of their relative frequencies, i.e. the number of articles that count as hits for a search term/ the number of *all published articles* in the journal.

To calculate the percentages for individual interaction terms it was necessary to estimate the total number of articles published in journals. I used the letter ‘a’ to search for hits with articles for each period investigated (a similar search method to the one described above). These numbers were recorded as estimations of the numbers of all articles published for each journal and for each decade investigated. I cross-checked some of the estimations by calculating the items in journals.

The ND values, CD values, and percentages for individual interaction terms are different bias measures. The percentages for individual interaction terms measure the frequency of an interaction term relative to *all the published topics* in a journal, whereas ND and CD values measure the proportion of negative interactions (ND) or competition (CD) to *topics that have to do with biological interactions* in a journal.

The reason for applying different bias measures for the same publication data set is that previous authors have utilized bias measures similar to ND and CD values (see section "Discussion"), which seems to support the old ideas. However, it is worthwhile examining whether other measures provide robust or different results and how reliable the results based on different measures are.

**Fig. 1:**
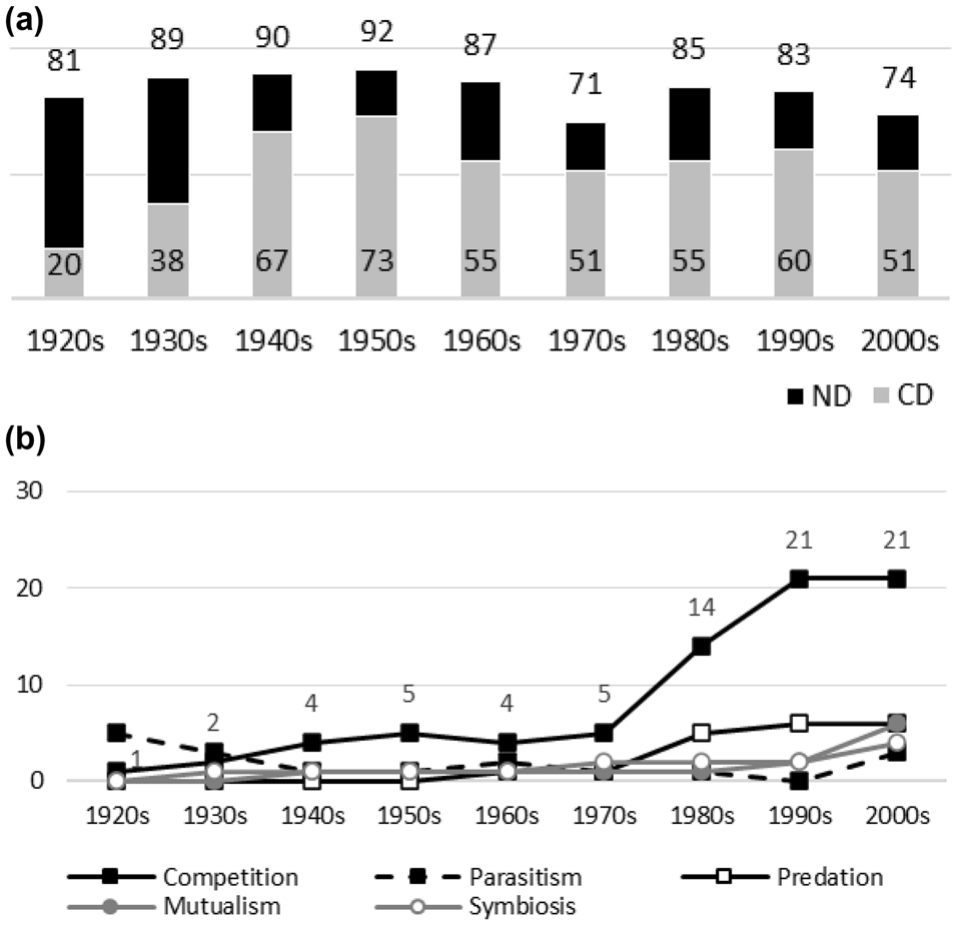
**a** ND (black bars) and CD (grey bars) values in the *American Journal of Botany*. Negative interactions dominate during the whole publication history. The highest ND value is reported during the 1950s, whereas the lowest one is reported in the 1970s. There are no clear temporal patterns in CD values: the earliest CD values are lower than later ones, whereas the highest CD values are reported during the 1940s and 1950s. **b** Percentages for interaction terms in the *American Journal of Botany*. The percentages for negative interaction terms are less than 10% during the early publication history (i.e. no biases are visible in data). The percentages for competition rise sharply after the 1970s and it becomes a dominant topic. There is a minor increase in the percentages for predation during the same periods. Other interaction topics remain marginal

**Fig. 2:**
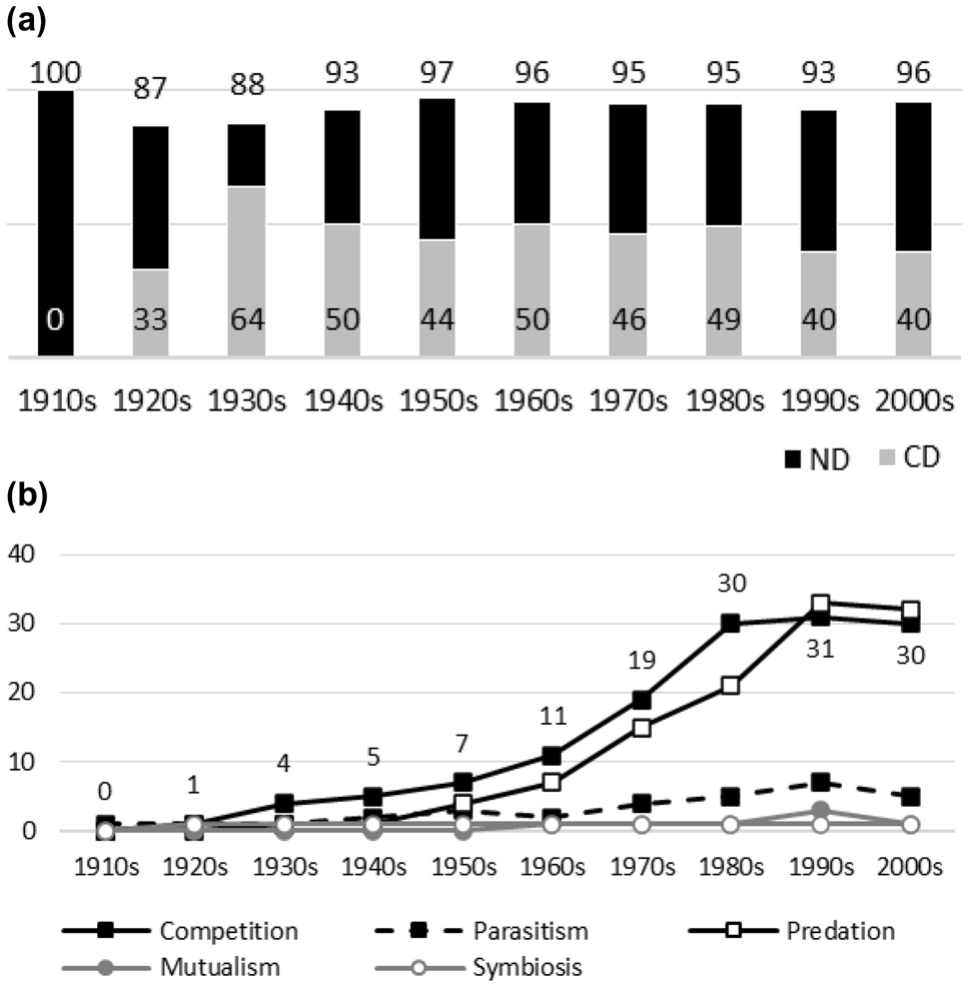
**a** *The American Midland Naturalist*. Negative interactions dominate during the whole publication history: the lowest ND value in the 1920s is just after the highest value. There are no clear temporal patterns in CD values. The lowest CD value is the earliest one. The highest CD value appears in the 1930s. **b** *The American Midland Naturalist*. Before the 1960s, no biases are visible in publication data. After this, the percentages for competition and predation rise sharply and the two become dominant research topics. Other interaction topics remain marginal

**Fig. 3:**
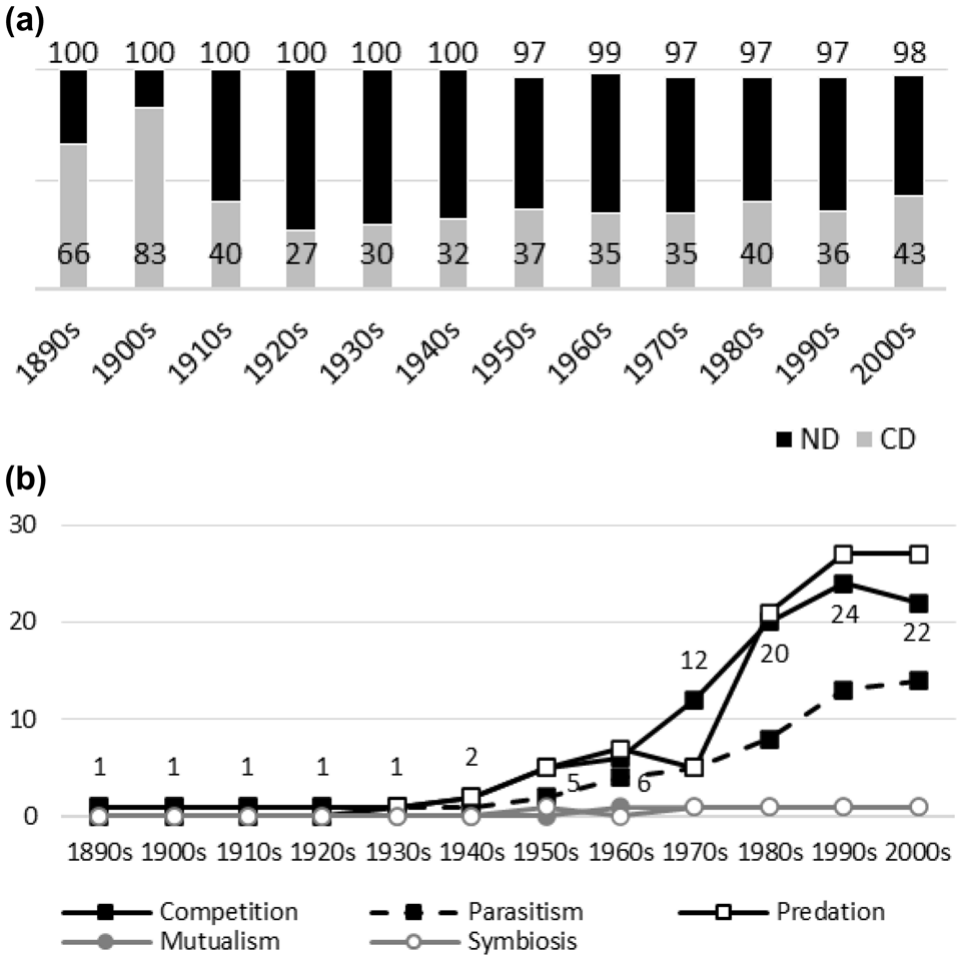
**a** *The Auk*. Negative interactions dominate at high levels during the whole publication history. Differences between the highest and lowest ND values are marginal. CD values suggest that the dominance of competition has diminished. The pattern is not monotonic. **b** *The Auk*. There are no noticeable biases in data before the 1970s, after which competition, predation, and parasitism become dominant. Positive interactions remain marginal topics

**Fig. 4:**
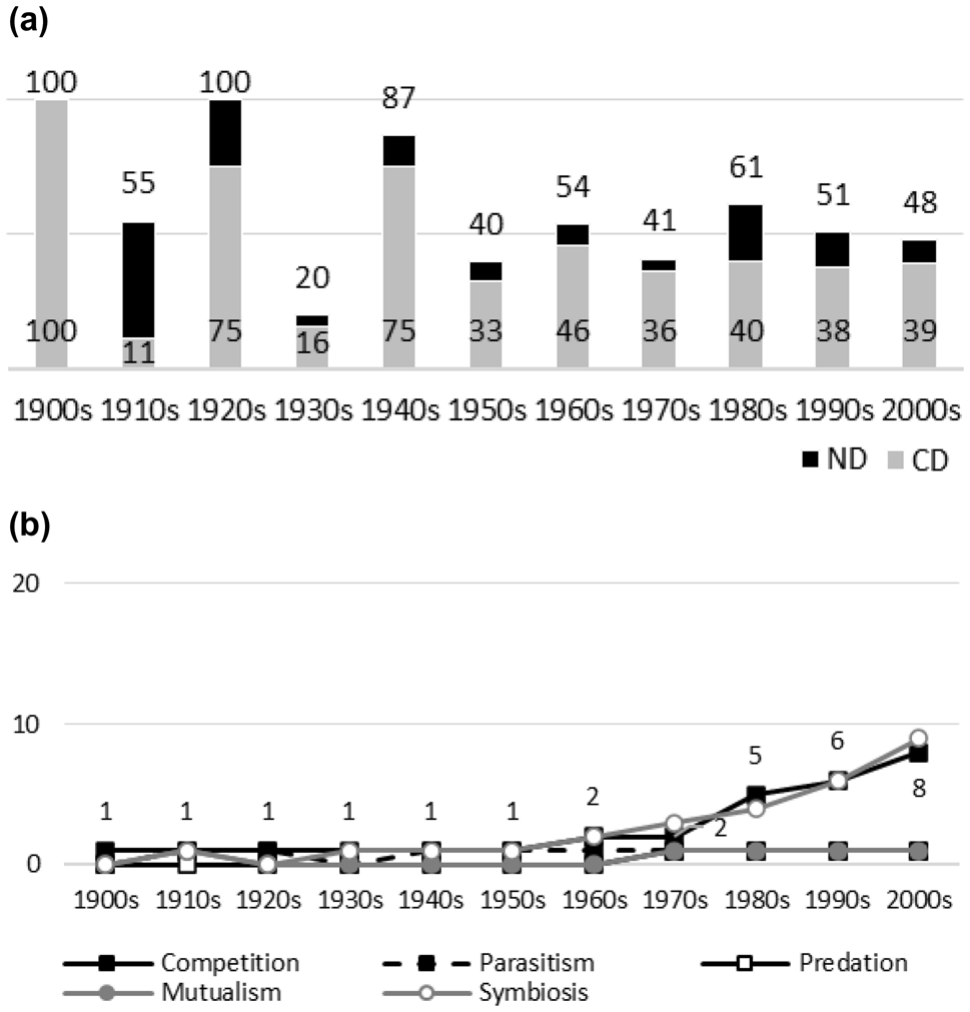
**a** *The Bryologist*. Early variance in ND and CD values is due to low numbers of articles published on interaction topics (see Table 4). More recent data suggest low or no dominance of negative interactions (many later ND values are less than 50%). However, CD values remain at relatively high levels even in the light of more recent data. There is data to suggest that the past dominance of competition has diminished (high early CD values with two exceptions). **b** *The Bryologist*. While there are no noticeable biases in data, the popularity of symbiosis and competition rise after the 1960s. Symbiosis seems to enjoy occasionally greater popularity as a topic than competition

**Fig. 5:**
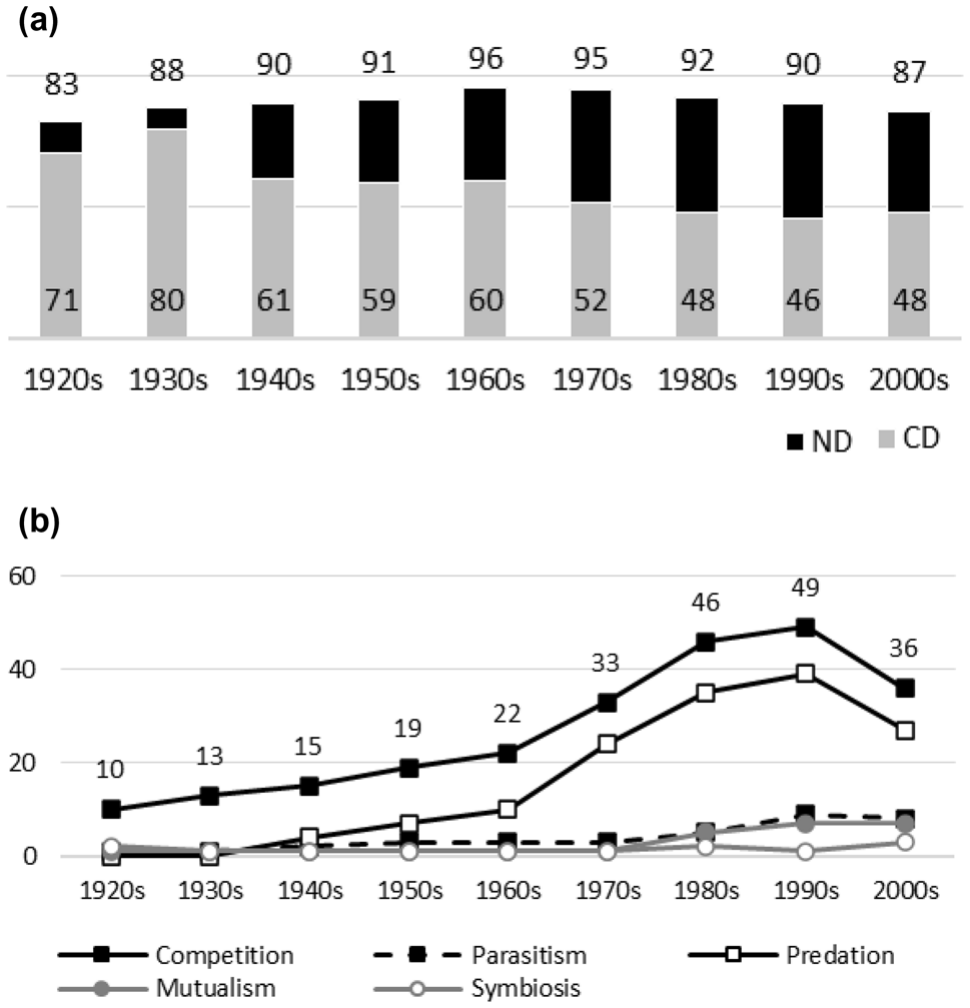
**a** *Ecology*. Negative interactions dominate during the whole publication history. Earlier CD values are generally higher than later ones, which implies that the dominance of competition has diminished. **b** *Ecology*. Competition has some historical popularity. Nevertheless, after the 1950s and/or the 1960s, the popularity of competition and predation rises sharply and they become dominant topics. While their popularity diminishes in the 2000s, the dominance does not disappear. Other interaction topics remain marginal

**Fig. 6:**
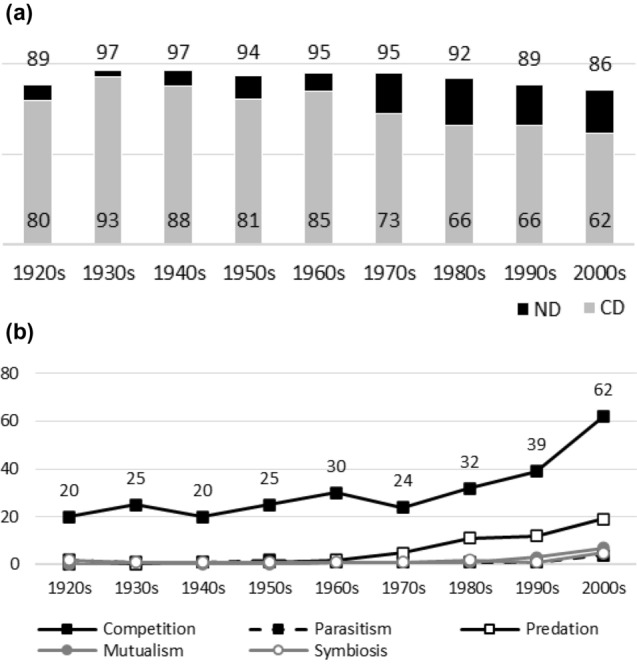
**a** *Journal of Ecology.* Negative interactions dominate during the whole publication history: the highest ND values appearing in the 1930s and 1940s and the lowest ND value is the most recent one. Earlier CD values are higher than later ones (the highest CD value is during the 1930s), which implies that the dominance of competition has diminished. **b** *Journal of Ecology*. Competition has historical dominance as a topic. But, as with other journals, this dominance increases with time. Other interaction topics remain marginal

**Fig. 7:**
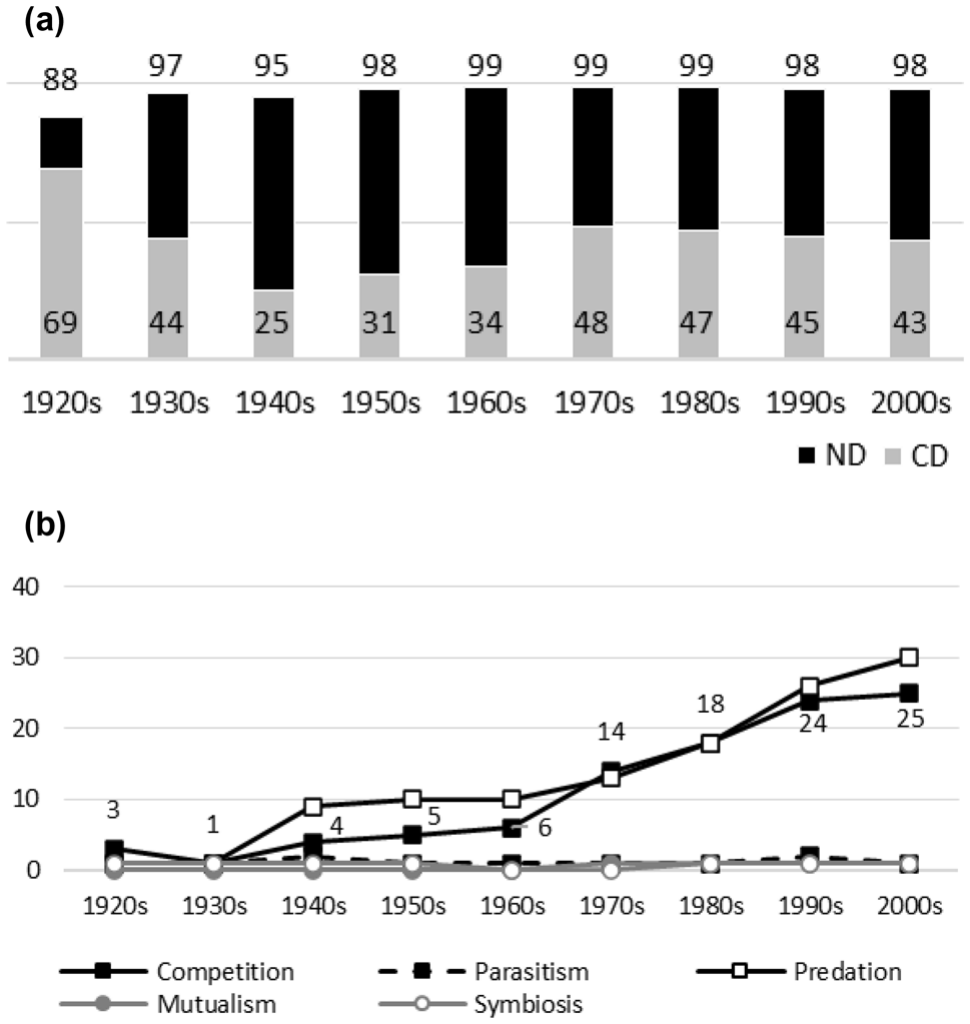
**a** *Journal of Mammalogy*. Negative interactions dominate during the whole publication history. Although the data suggest that the dominance of competition has diminished, this is partly due to a high CD value in the 1920s. **b** *Journal of Mammalogy.* The popularity of competition and predation rises after the 1960s and the two establish themselves as dominant research topics after this

**Fig. 8:**
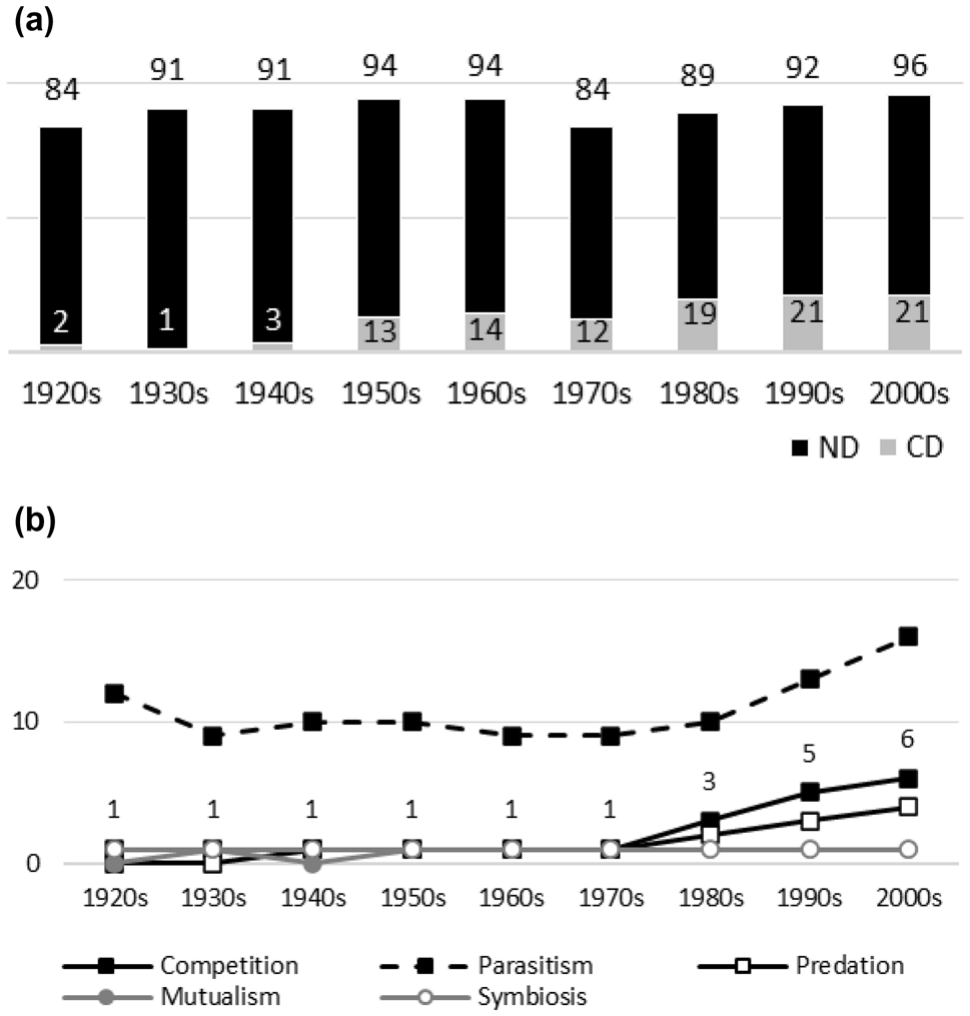
**a** *Journal of Parasitolo*gy. Negative interactions dominate during the whole publication history: the highest ND value being the most recent one and the lowest values appearing in the 1920s and 1970s. The dominance of negative interactions is due to other negative interactions than competition (low CD values). Nevertheless, the temporal pattern in CD values is the opposite of the past dominance of competition: CD values rise during the publication history. **b** *Journal of Parasitology*. The popularity of ‘parasitism’ proved to be lower than anticipated. Parasitologists might consider the term redundant in the context and/or utilize more diverse terminology. As with almost all the other journals investigated, the popularity of competition and predation rises after the 1970s, even though the two remain marginal as topics even after this. Positive interaction topics remain stably marginal

**Fig. 9:**
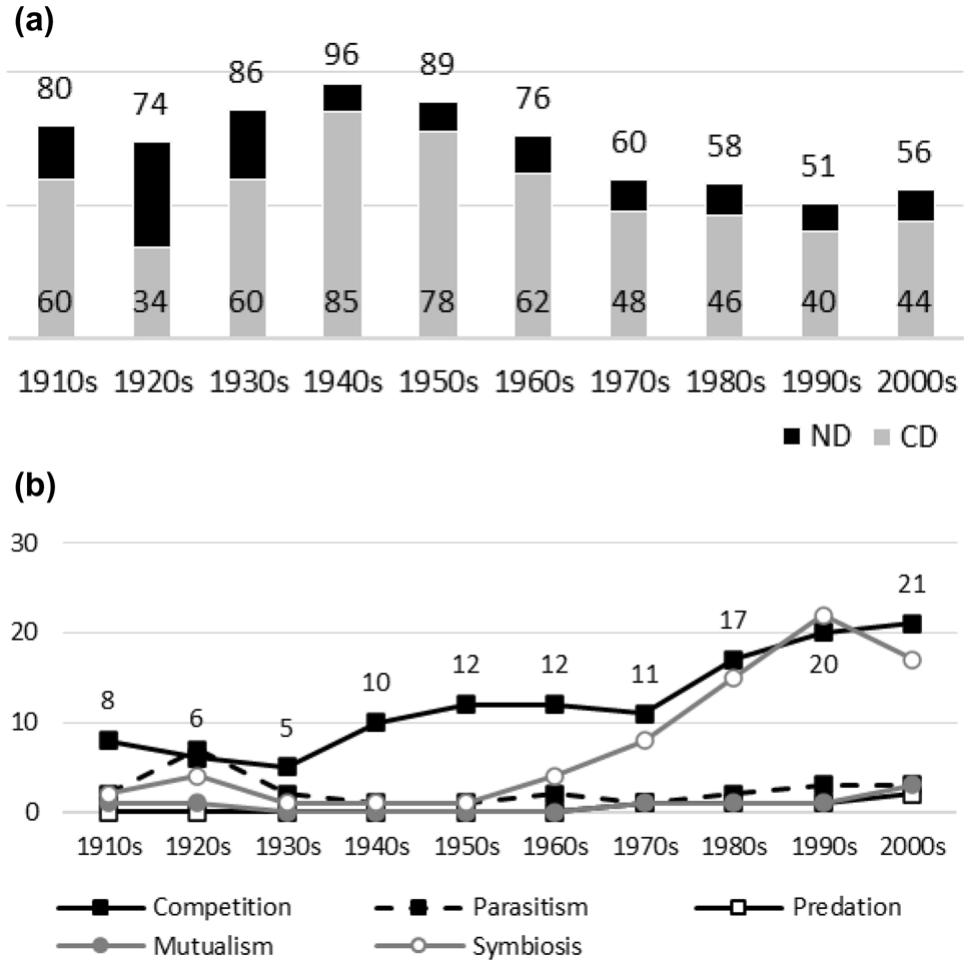
**a** *New Phytologist*. ND values are high(er) during the early and middle publication history. After this, ND values diminish. The pattern is not monotonic. The CD values suggest that the dominance of competition has also diminished. This pattern is not without exceptions either. **b** *New Phytologist*. Competition has some earlier popularity (notice also the peak for parasitism in the 1920s). Its more dominant position is established, however, during the 1980s and 1990s. The percentages for ‘symbiosis’ rise sharply after the 1960s. During the 1990s, the popularity of symbiosis is higher than that of competition

**Fig. 10:**
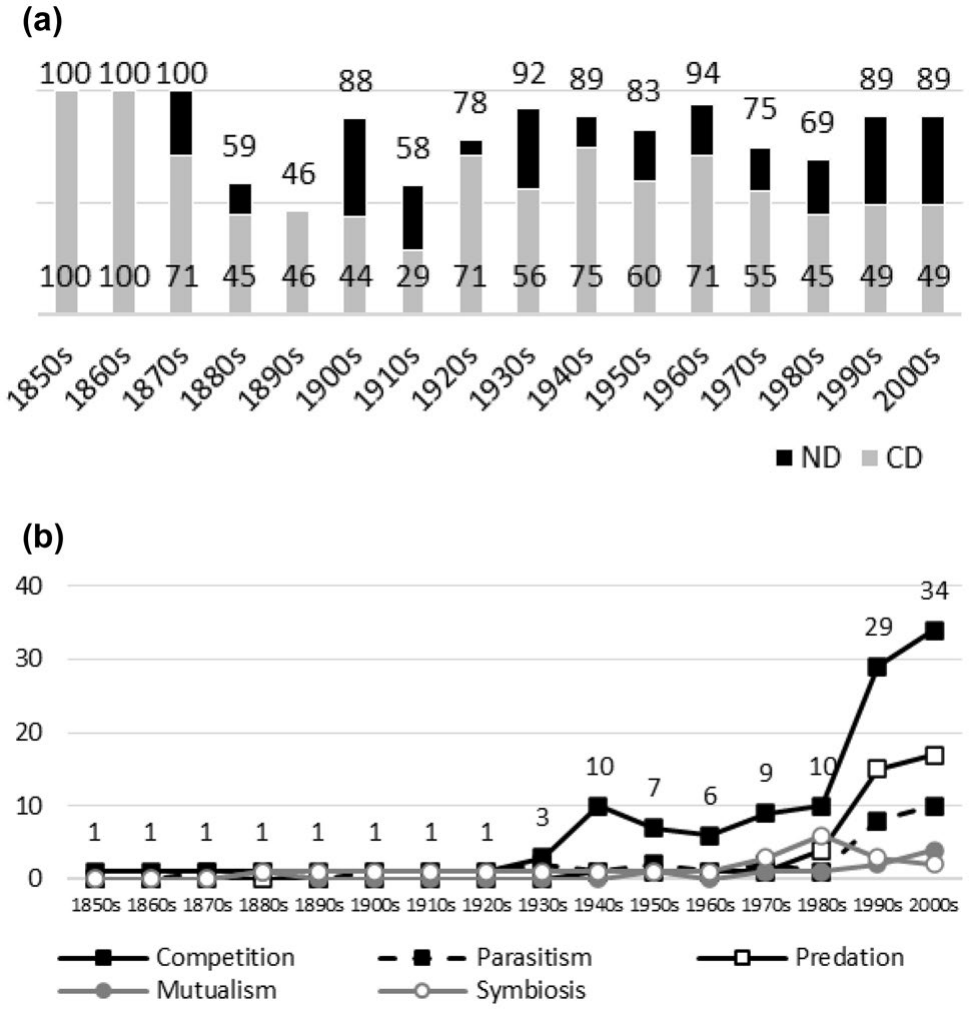
**a** *Proceedings of the Royal Society of London. Series B, Biological Sciences*. Early variance in ND (and CD) values is due to the low number of published articles on interaction topics (see Table 10). ND values suggest the high dominance of negative interactions, although there are exceptions. The temporal pattern in CD values suggests a diminution in the dominance of competition: the latest CD values are lower than most of the earlier ones. The pattern has exceptions. I have omitted data before the 1850s from Figs. 10a and 10b. In practice, JSTOR provided no hits for interaction terms before the 1850s. This was partly expected. Some of the terms, such as symbiosis, mutualism, and commensalism, were coined during the 1870s. **b** *Proceedings of the Royal Society of London. Series B, Biological Sciences.* Interaction topics remain marginal up until the 1940s, after which competition gains popularity. It is only after the 1980s when the popularity of competition rises sharply (the percentages for predation and parasitism rise as well, but to a lesser extent). Positive interactions are marginal research topics

**Fig. 11:**
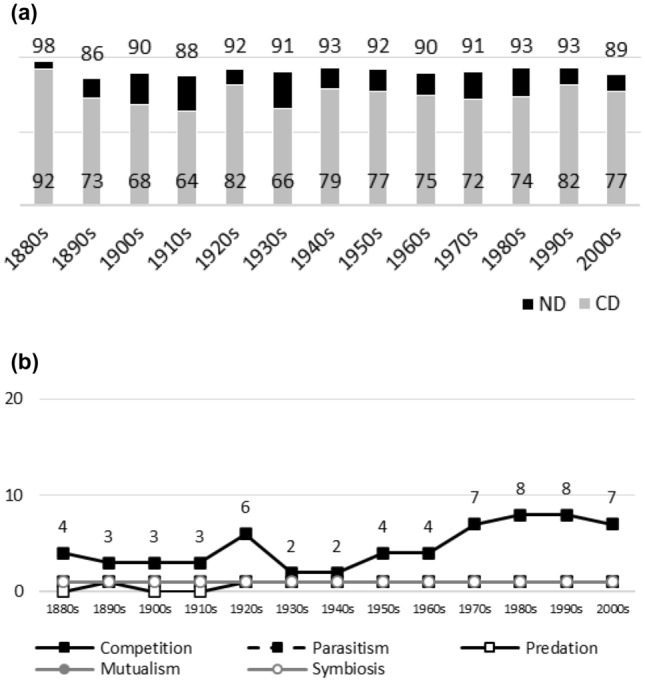
**a** *Science.* Negative interactions dominate during the whole publication history. There are high CD values during the whole publication history. **b** *Science.* Interaction topics remain marginal during the whole publication history (less than 10% for all terms). Nevertheless, there is a rise in the popularity of competition after the 1960s, which happens during the same time as in biology journals. The reasons for the popularity peak of competition in the 1920s are unclear (the topics in the articles cover a variety of biological phenomena)

Since the object is to provide long-term data on biases in biology, data sources must have long publication histories. JSTOR’s database covers over 160 biology journal titles. Unfortunately, the great majority of titles have short publication histories (e.g. 20–40 years). The following journals were selected as data sources, since, besides being major and respected journals from different biological disciplines, all have data available since at least 1920: the *American Journal of Botany* (Fig. 1a, b), *The American Midland Naturalist* (Fig. 2a, b), *The Auk* (Fig. 3a, b), *The Bryologist* (Fig. 4a, b), *Ecology* (Fig. 5a, b), the *Journal of Ecology* (Fig. 6a, b), the *Journal of Mammalogy* (Fig. 7a, b), the *Journal of Parasitology* (Fig. 8a, b), the *New Phytologists* (Fig. 9a, b), the *Proceedings of the Royal Society of London. Series B, Biological Sciences* (Fig. 10a, b), and *Science* (Fig. 11a, b).

Ten-year periods (full decades, such as 1920–1929) were chosen as the interval for the collection and comparison of data.

Figures 1, 2, 3, 4, 5, 6, 7, 8, 9, 10 and 11 visualize the data. Figures marked ‘a’ visualize ND and CD values (section "ND and CD values"), and figures marked ‘b’ visualize the percentage data for individual interaction terms (section "Percentages for interaction terms"). As amensalism and commensalism stay steadily at low percentages (0 – < 1%), I have omitted both from the ‘b’ figures. The only numbers I rounded up are the percentages ‘less than one’ for individual interaction terms in figures b: many early values are ‘ < 1%.’ The number of hits data for each interaction term and the number of all published articles in each journal/decade are reported in Tables 1, 2, 3, 4, 5, 6, 7, 8, 9, 10,  11 (see Appendix).

Data on interaction terms were collected in December 2020 and January 2021 with the exception of the *New Phytologist*. In this case, I used Google Scholar, since in June 2021 JSTOR failed to function properly due to an update. Additionally, I cross-checked the hits for interaction terms of this study by using Google Scholar for some of the journals and periods (June 2021). The Google Scholar results were both consistent with and similar to the results of JSTOR. For this study, JSTOR was the more convenient option, as there the estimations of the number of articles published in different journals seem to be closer to their true values than on Google Scholar. Moreover, the narrowing down of data sources is more accurate and reliable when using JSTOR. Despite the fact that the results seem to be robust in that different and independent search engines/algorithms provide similar results, the results reported are evidence of general trends only, since all search engines deliver both false positive and false negative hits.

Data sources include both general and specific topic journals from different disciplines within biology. *Science* was included to examine whether a general topic science journal differs in data from biology journals. Although the sample size (*n* = 11) is small, adding further data sources would be redundant. First, additional sources would be either plant biology or general topic science journals, which are already included. Second, increasing the sample size would not add anything qualitatively new, since the results are clear and robust enough with the current sample size.

## Results

In this section, the results between ND and CD values (section "ND and CD values") and the percentages for individual interaction terms (section "Percentages for interaction terms") are compared. When these measures are applied to the same publication data, they provide conflicting results concerning the biased nature of biology, especially in the past. This is mainly since the ND and CD values measure the biases within interaction topics, whereas the percentages measure the biases within all the topics, including non-interaction topics.

Since the old narrative is based on the ideas of past biases and subsequent progress in correcting for them, bias measures should display that the biased nature of biology diminishes with time. In other words, bias measures should display that the popularity of a certain historically dominant topic(s) decreases with time, whereas the popularity of alternative and historically more marginal topics should increase with time. Since another popular idea among biologists is that modern biology was biased from its beginning (see section "Discussion"), we can presume that past biases should be visible early in journals’ publication histories as well.

### ND and CD values

A bias towards negative interactions should be visible in a journal’s ND values, whereas a bias in competition should be visible in its CD values. A 70% ND value and a 30% CD value are suggestions for the lower limits of biases.^3^

Were the old ideas true, ND and/or CD values should diminish with time during a journal’s publication history, since this indicates that the biases were higher in the past (i.e. past biases have been corrected). However, due to external or confounding factors (e.g. low numbers of articles published on interaction topics during a period, or changes in editors or the journal’s scope), ND/CD values do not always mirror the research interests of a journal’s scientific community (e.g. botanists or ecologists). Let us allow that the temporal patterns in journals’ diminishing ND or CD values need not to be monotonic. A few exceptions must be allowed in the case of individual journals.

Generally speaking, ND values stay at 80 + % levels in journals during their whole publication histories. ND values less than 80% are the exception (cf. Figures 4a, 9a, 10a). The complete dominance of negative interactions is not uncommon (cf. Figures 2a, 3a, 4a, 10a). These 100% values seem to be early ND values.

Even though the complete dominance of negative interactions appears early in journal publication histories, there seem to be no clear temporal trends in ND values. Rather, journals have their lowest and highest ND values during different periods of publication history (cf. Figures 1a, 2a, 3a, 4a, 5a, 6a, 7a, 8a, 9a, 10a and 11a). The two exceptions are the *New Phytologist* (Fig. 9a) and *The Auk* (Fig. 3a) in which ND values seem to be higher in the past.

Negative interactions seem to dominate specific topic biology journals (Figs. 6a, 8a^4^), general topic biology journals (Figs. 2a, 5a), and even general topic science journals (Fig. 11a).

The general implication of ND value data is that there *is* evidence for *past biases* in biology, but *no* evidence that these were *higher* in the past. There are no clear temporal patterns in the journals’ ND values, such as later ND values being lower than earlier ones, apart from the two exceptions mentioned above. Since ND values are generally speaking over 80% during the whole publication histories of different journals (with the exception of Fig. 4a), this implies constant high levels of biases in biology vis-à-vis negative interactions.

What ND value data show is that the less popular version of the old ideas (the past dominance of negative interactions) is not true [contra (May 1982; Keddy 1990; Bronstein 2009)]. Notice that ND value data are compatible with the more popular version of the old ideas, which are about the historical dominance of *competition* [e.g. Kropotkin (1890a, 1890b), Risch and Boucher (1976), Lewin (1983), Cherif (1990)].

A few instances of complete dominance of competition can be found in the journals’ CD value data (Figs. 4a, 10a). These 100% values are also early ones. Moreover, in seven journals there is evidence that earlier CD values are higher than later ones. The evidence is clearer in some journals (Figs. 3a, 5a, 6a) than in others (Figs. 4a, 7a, 9a, 10a).

The temporal pattern of CD values in *Science* (Fig. 11a) is difficult to interpret expect that competition has enjoyed high popularity during the whole publication history.^5^ The *American Journal of Botany* (Fig. 1a) and *The American Midland Naturalist* (Fig. 2a) present no clear temporal patterns in CD value data. The *Journal of Parasitology* (Fig. 8a) presents contrary evidence that competition dominated more in the past as its CD values rise with time.

While temporal patterns of CD values are not monotonic nor without exceptions and while evidence is clearer in the case of certain journals than in others, generally speaking CD value data can be taken to suggest that competition has dominated biological research more in the past. There are high past CD values (over 60%) in many journals and these values are generally speaking higher than the later CD values in journals. That is, the data seem to indicate a higher past bias of competition in biology.

ND and CD value data together imply that *other negative interaction topics* have become more popular as the historical dominance of *competition* has diminished with time. ND and CD value data are not only compatible with the more popular version of the old ideas but seems to give evidential support to it as well. There seemed to be a heavy emphasis on competition in the past, as high early CD values in many journals suggests. That is, the idea that there was a past bias in biology seems to be confirmed. There seems to have been subsequent progress as well, since biologists have started to focus more on other negative interaction topics rather than competition, such as predation and parasitism, which is the implication of constant ND values in journals, when journals’ CD values are diminishing with time. That is, the other idea is apparently also confirmed, since the popularity of a historically dominant topic (competition) has decreased with time, whereas the popularity of alternative, more marginal topics (parasitism and predation) has increased with time, which suggests that there has been subsequent progress in correcting past biases in biology.^6^

### Percentages for interaction terms

Ten per cent popularity is suggested as a bias threshold for an individual interaction term, i.e. below the 10% threshold, no bias exists with regard to an individual interaction term. Given the diversity of *non*-interaction topics in biology, the threshold is conservative.^7^

When the popularity of interaction topics is measured in terms of the percentages for individual interaction terms and relative to all the published topics, a different picture of biases emerges than the one suggested by ND values or CD values, which measures biases relative to interaction topics only.

For instance, journals that seem to be very biased in terms of ND and/or CD values, such as the *Proceedings* (Fig. 10a) and *Science* (Fig. 11a), are not biased when the percentage data for individual interaction terms is investigated (Fig. 10b and 11b). In both, negative interactions, including competition, are marginal as research topics except for the two last decades of the *Proceedings*.

The percentage data for individual interaction terms, moreover, display that the biased nature of biology increases with time, in contrast to what the CD value data (higher biases in the past) and the ND value data (constant biases in biology) suggested. This pattern is visible in almost all the journals investigated. In certain cases, the popularity of competition rises sharply with time (Figs. 1b, 4b, 6b, 9b, 11b). In other cases, the popularity of competition and predation (and sometimes parasitism) rises sharply with time (Figs. 2b, 3b, 5b, 7b, 8b, 10b).

Negative interaction topics have become abruptly dominant recently. Their popularity typically rises sharply during the 1960s or 1970s in journals. Before that, interaction topics seemed to be marginal topics. Moreover, there is hardly any data for the dominance of competition in the past, when the popularity of competition is measured against all the topics published in journals, in contrast to what the CD values suggested. A putative exception is the *Journal of Ecology* (Fig. 6b). Nevertheless, even in this case, as in other journals, the pattern is the opposite to the traditional ideas, since the bias (i.e. the popularity of competition) increases with time.

The current rise in the popularity of negative interaction topics affects even journals that one might expect to remain unaffected. In the *Journal of Parasitology* (Fig. 8b), competition and predation remain very marginal up until the 1970s, as might be expected given the title. After this, their popularity rises, as in other biology journals. In *Science* (Fig. 11b), competition remains marginal up until the 1960s, after which its popularity rises. This establishes that the sharp rise in the popularity of negative interactions is a general phenomenon in the scientific community.

## Discussion

When biologists investigate the past trends or biases in biology, they often utilize bias measures similar to ND or CD values. Authors compare the popularity of negative interaction topics, especially competition and sometimes predation, to positive interaction topics, without paying attention to how popular interaction topics are relative to all published or discussed topics in the literature, articles, or textbooks [cf. Risch and Boucher (1976: 8), May and Seger (1986: 260), Keddy (1990: 101), Martin and Schwab (2013: 35–36)].

One explanation why different authors have (re-)discovered the old ideas is that they have utilized bias measures similar to ND or CD values. ND and CD values seem to provide us with evidence for the ideas of past biases in biology and for subsequent progress in correcting them. The joint implication of ND and CD value data was that while the level of biases regarding negative interactions has been constant in biology, the nature of the bias has changed. Other negative interactions have become more popular as the popularity of competition has diminished with time (for diminishing CD values in journals, but constantly high ND values, see section "ND and CD values"). ND and CD values seem to confirm the old ideas.

However, the main problem with ND and CD values is that they give a strong impression of biases even if the measured topics are marginal research topics. This is because ND and CD values only measure biases within interaction topics.

The percentage data for individual interaction terms (section "Percentages for interaction terms") show that the evidence for the idea of high(er) past biases is spurious. *Within* the interaction topics, it is true that competition may have enjoyed popularity even in the past. It may have even dominated interaction topic research in the past, as CD values data in section "ND and CD values" show. Nevertheless, as data in section "Percentages for interaction terms" indicates, competition was a marginal research topic in the past, when its popularity is measured against all the published topics. The same is true of negative interaction topics in general, in contrast to what ND values suggested in section "ND and CD values". A bias in a marginal topic is not a bias of and in biology. Thus, instead of there being a past research bias in biology, a more accurate description of the situation might be that the past emphasis on competition within interaction topics represented a research oddity or peculiarity.

Negative interaction topics, including competition, become mainstream and dominant after the 1960 and 1970s, as the data in section "Percentages for interaction terms" shows. In fact, the percentage data for individual interaction terms suggest that the idea of the biased past of biology is false. Precisely the opposite is true: the popularity of competition and other negative interactions *increases with time*, the past being free of biases. ND and CD values are not only unreliable but are misleading bias measures.

Where does the idea of the past biased nature of biology come from besides bias measures that are similar to ND and CD values? The obvious candidate is from the writings and readings of Darwin:

[S]tudents of evolution, genetics, and ecology derived their ideas of, and interest in, competition largely from *On the Origin of Species*. In it, Darwin described competition as universal (1859, p. 60) and as the chief component (pp. 205, 220) of the struggle and of natural selection. “Compete”, “competition”, and “competitor” are used eighty-one times in the *Origin*,^8^ sometimes in conjunction with the familiar phrases “struggle for life”, “struggle for existence”, and the equally loaded words “battle” and “war” (McIntosh 1992: 61).

Equally common is the idea that Darwin inherited this overemphasis from Malthus, political economy, and/or the Victorian era:

Although Malthus’ influence must be considered paramount, it seems that the very ambiance of the Victorian age – more particularly its competitive ethos – contributed substantially to Darwin’s strong emphasis on struggle and conflict in the *Origin* (Gale 1972: 343).

From Thomas Malthus and Adam Smith to Charles Darwin and Herbert Spencer, the idea of competition began to be recognized as an important factor in nature… Since the 19th century human progress through competition has become the dominant theme in both the natural and social sciences (Cherif 1990: 206).

The idea that there was an overemphasis on competition in Darwin, which he inherited from Malthus and/or the Victorian Zeitgeist has been popular, especially among biologists (Risch and Boucher 1976: 8–9; Boucher 1985: 9–11; Bruno et al. 2003: 119; Holt 2009: 2–3; Lewontin 2009: 20). The implication seems to be that modern biology was destined to be biased from the start. Another implication one gets from ideas such as these is that interaction topics were dominant or at least popular topics among naturalists even during the Victorian era. This, in turn, might explain why biologists have utilized unreliable measures, such as ND and CD values. If interaction topics were popular topics even in the past, there is no need to measure the biases within all the published topics. This is a false as an assumption.

The idea that there was an overemphasis on competition in Darwin and/or among contemporary Occidental naturalists, derives typically from the writings of mutual aid theorists and their readings of Darwin. Mutual aid theory arose after the publication of the *Origin* in Russia as a reaction to Darwin’s presumed overemphasis on competition (or “struggle”). Mutual aid theory emphasized positive interactions and climate rather than competition as factors in evolution.The best-known of mutual aid theorists was Kropotkin.^9^ The following passage illustrates the ideas concerning the past biases in biology (overemphasis on intraspecific competition) and the subsequent progress in correcting them (positive interactions should be given equal emphasis):

The readiness of the Russian zoologists to accept Kessler’s views [on the importance of mutualistic interactions, when organisms struggle with abiotic factors] seems quite natural, because nearly all of them have had opportunities of studying the animal world in the wide uninhabited regions of Northern Asia and East Russia… . I recollect myself the impression produced upon me by the animal world of Siberia, when I explored the Vitim regions in the company of so accomplished a zoologist as my friend Polyakoff was. We were both under the fresh impression of the *Origin of Species*, but we vainly looked for the keen competition between animals of the same species which the reading of Darwin’s work has prepared us to expect. … [W]e witnessed numbers of facts of mutual aid… but even in the Amur and Usuri regions, where animal life swarms in abundance, facts of real competition and struggle between higher animals of the same species came very seldom under our notice, though we eagerly searched for them. The same impression appears in the most of Russian zoologists, and it probably explained why Kessler’s ideas were so welcomed by the Russian Darwinists, whilst like ideas are not in vogue in the followers of Darwin in Western Europe (Kropotkin 1890a: 341–342).

Kropotkin’s (1890a, 1890b) original argument against the claimed overemphasis on competition was in line with Darwin’s theory presented in the *Origin*. He described observed cases of mutualistic interactions in various taxa, especially in birds, to show that such cases outnumber those in which individuals compete in nature. In addition, he tried to establish that mutual aid confers a higher fitness than competitive abilities, and finally he tried to show that climate rather than competition is the main factor responsible for keeping population numbers in check in nature. Later, Kropotkin (1910) distances himself from Darwinism when he resorts to the then fashionable neo-Lamarckism. The claim was that the inheritance of acquired characteristics and the direct impact of the environment, rather than competition, were enough to explain adaptations, speciation events, and so on. Notice that Kropotkin does not here necessarily distance himself from Darwin. After the first edition of the *Origin*, Darwin began to show more interest in neo-Lamarckism, a fact that Kropotkin (1910) cleverly capitalizes on.

I shall not take any position on whether Darwin overemphasized competition, as Kropotkin (ibid). and many biologists have suggested. Darwin (1859: 62–63) warned against reifying the metaphorical “struggle” with competition. Moreover, while the influence of Darwin’s writings cannot be denied, it seems misplaced to attribute to Darwin [cf. McIntosh (1992) above] a fact that is manifested in biology one hundred years after the publication of the *Origin*, a fact that manifested itself also *differently* from what the authors suggest (i.e. in most of the journals investigated in section "Percentages for interaction terms", negative interactions dominate during and after the mid-twentieth century, not competition alone).

Another dubious aspect of the above attributions of past biases to Darwin concern Malthus’ influence. Despite Darwin’s self-claimed debt to Malthus, evidence that Malthus was responsible for Darwin’s emphasis on competition is unclear. First, it is not clear what precise influence Malthus had on Darwin’s ideas besides the general notion of overpopulation, which was in any case a popular cultural idea (Oldroyd 1984). Second, the concept of ‘competition’ that political economists, including Malthus, utilized was different from the zero-sum conception that Darwin and other biologists used (Gordon 1989). It is ironic that mutual aid theorists accused Darwin of using a Malthusian concept of competition [see Todes (1987) for references and discussion]. Political economists seemed to understand competition as a positive interaction term. In biology we might call such competition mutualism. The irony is that had mutual aid theorists been correct in their accusations, Darwin would have based his theory of natural selection on mutualistic interactions and thus would been their ally.

If there is no clear evidence that there was an overemphasis on competition in Darwin’s writings, what about the idea that the past overemphasis on competition in biology can be attributed to and ultimately derives from Victorian era/science, as many authors have suggested [e.g. Gale (1972), Risch and Boucher (1976)]. Compare Fig. 10b in section "Percentages for interaction terms", which reflects science during the Victorian era. The *Proceedings* was one of the flagships of British scientific journals. Typically, less than one per cent of published articles referred to competition between 1850 and 1929 in the *Proceedings*. The proportions of articles in the *Proceedings* between 1850 and 1889 should in fact be expressed in terms of ‰s for competition (cf. Table 10). Moreover, many of the hits for ‘competition’ during this era deal with social and economic rather than biological phenomena in the journal.

Although the *Proceedings* started as a general topic science journal and was split into two series, A and B, in 1905, an examination of articles published before the split reveals that biological topics from anatomy to parasites were well represented. That is, the lack of a bias concerning competition is not due to the neglect of biological topics in the journal. Moreover, in the 1910s data no noticeable trend towards an overemphasis on competition is visible in Fig. 10b, even though the B series is now devoted to biological matters.^10^ There is simply no bias towards competition in the past in the *Proceedings* that is visible in its publication data. Evidence for past biases of other negative interactions, such as predation, are even harder to find in publication data. The bias pattern in the *Proceedings* towards competition is the opposite to its earlier dominance, namely a high and abrupt recent bias.The same pattern is visible in other journals investigated in section "Percentages for interaction terms".

There thus seems to be no data that there was an overemphasis on competition in Victorian biology, in contrast to what many authors have suggested. Rather than there being an overemphasis, there seems to be an underemphasis on competition in Victorian biology.

The conclusion is that popular ideas concerning the past biases and subsequent progress in correcting them are myths. Evidence for the ideas comes either from unreliable and misleading bias measures, such as ND and CD values, or from controversial readings of Malthus, Darwin, and Kropotkin. In both cases, the same mistake is committed: evidence is sought from sources that are not only most amenable to displaying the biases but also overinflate their importance.

Several biologists have expressed the ideas of the biased past of biology and progress in one way or another. I will next focus on a few examples from the 1980s and 1990s, as during this period it clear that biology has become biased.^11^ Does this mean that the ideas are no longer myths and the authors are correct? No.

The opening of May’s (1982: 803) article on mutualistic interactions illustrates the ideas:

Mutualistic interactions between species have traditionally received less attention from ecologists than competitive and predator–prey interactions. Recent years have, however, seen the growing awareness of this fact, and, in my opinion, empirical and theoretical studies of mutualistic associations are likely to be one of the growth industries of the 1980s.

May is wearing more than one hat here. Several authors have blamed May’s (1973) theoretical results in the context of analytic population models, namely, that mutualistic interactions lead to unstable communities, among the main modern reasons for the biases in competition (and predation) in biology (e.g. Keller 1992).^12^ May mentions that a lack of “stability” is one of the main reasons for the paucity of mutualism studies. This leads to a hybrid position: May is trying to provide justifications for the past biases and at the same time suggests that we should try to correct them once they have been noticed.

In contrast to May, Boucher et al. (1982: 318) state that the reason why mutualism has been neglected is due to ideological reasons:

[M]utualism has been avoided during most of the 20th century because of its association with left-wing politics (perhaps especially with Kropotkin).

While Kropotkin’s political ideology might have contributed to his version of mutual aid theory (cf. Kinna 1995), arguments connecting the lack of mutualism studies with “leftist” political ideas are hasty.^13^ First, mutual aid theorists held diverse political views (see Todes 1987). Second, in addition to studying de-populated harsh artic environments, national *cultural ethos* emphasizing communal values might have contributed to the development and acceptance of the mutual aid theory in Russia. Traditional ideas, such as ‘obshchina’ and ‘mir’ (cf. Grant 1976), were projected onto nature with the consequence that mutualistic interactions in natural societies become an important research topic, whereas self-interested competitive individuals were viewed as outcasts. While the English translation of both obshchina and mir is ‘commune,’ they are value-laden concepts that emphasize the collective values and virtues of idealized Slavic peasant communities, such as cooperation (i.e. mutualism).Finally, it could be that mutual aid theory—and to a certain extent mutualism—went out of fashion due to non-political reasons: Kropotkin (1910) made a wrong, and in retrospect, unnecessary, move when he connected mutual aid theory with neo-Lamarckism. This might have contributed to the view that the theory was regarded as outdated after Darwin’s theory and Mendelian genetics were synthesized. This is unfortunate, since Kropotkin’s (1890a, 1890b) mutual aid theory would have been compatible with this synthesis.

Another expression of ideas comes from Lewin (1983: 737):

For more than two decades the phenomenon of competition between species has been prominent—and some say completely dominant—in ecologists’ thinking about the way communities are shaped. Indeed, as Jonathan Roughgarden, of Stanford University, puts it, “Competition theory was the only theory on the block.”

Lewin (1983: 738) adds that “[t]he supposed dominance of interspecific competition and its putative constancy are both under challenge,” by which he anticipates that predation and abiotic factors are going to become more popular in the future. Thus, Lewin is repeating the old story: ecologists have noticed the past biases and are in the business of correcting them.^14^

In a similar manner, Keddy (1990: 101) notices the heavy traditional emphasis on competition (and predation) and the lesser emphasis on mutualistic interactions and suggests that the reasons for the past biases might be extra scientific, meaning that we need to correct these biases in order to progress:

[W]ith respect to research in ecology, we may be projecting our own cultural biases^15^ upon nature rather than studying forces in relative proportion to their importance in nature itself… . As ecology moves into the 1990s it is surely important to rectify this by choosing research questions and strategies according to objective criteria.

More recent authors have thus continued the narrative, which can be traced back to mutual aid theorists: a past bias is claimed to have been detected after which means are suggested how to correct for them. There are differences as well. The debate has shifted from evolution to ecology.^16^ Some recent authors have noticed that biology is biased not only with regard to competition, but also with regard to predation as well. Lewin’s (1983) argument is similar to Kropotkin’s except that predation has been substituted with mutualism. Other authors have been more sympathetic to mutualism recently, even though they have neglected abiotic factors, which mutual aid theorists deemed important. However, the most important difference between old and more recent narratives is that during the 1980s and 1990s biology has finally become biased. Not much has happened to “rectify” the situation. The biases were once again, as always, higher in the *future*. Consequently, the old ideas of past biases and the progress in correcting them remained myths even in the case of recent authors.

## Conclusions

Publication data on interaction topics suggest that two popular ideas among biologists concerning the biased past of biology and subsequent progress in correcting for the biases are myths. Rather than progress, the data suggest an abrupt recent regress in biology in that negative interactions have become dominant research topics in journals. Especially popular has been the myth concerning the past heavy emphasis on competition in (Victorian/Darwinian) biology. This myth should be dispensed with. None of the journals investigated displayed proper and reliable data on past biases of this sort. Moreover, in all the journals the popularity of competition rises with time. If anything, the data show that there was much *less* emphasis on competition in the past.

## Data Availability

Not applicable.

## Appendix

See Appendix

**Table 1:** American Journal of Botany

	Amensalism	Competition	Parasitism	Predation	Commensalism	Mutualism	Symbiosis	All articles
1920s	0	12	37	0	1	0	10	641
1930s	0	26	35	0	0	0	7	1000
1940s	0	54	18	0	0	1	7	1174
1950s	0	72	19	0	0	1	6	1358
1960s	0	70	39	1	0	3	13	1635
1970s	0	87	20	15	0	14	34	1610
1980s	1	293	34	120	1	33	42	2082
1990s	0	446	42	133	0	63	58	2111
2000s	0	495	72	146	0	145	103	2280

Number of hits for interaction terms and the number of all published articles

Tables 1,

**Table 2:** The American Midland Naturalist

	Amensalism	Competition	Parasitism	Predation	Commensalism	Mutualism	Symbiosis	All articles
1910s	0	0	5	0	0	0	0	305
1920s	0	3	4	0	1	0	1	229
1930s	0	40	11	4	4	0	3	807
1940s	0	61	31	20	2	0	6	1141
1950s	0	65	35	42	2	0	2	886
1960s	0	120	32	77	4	2	3	1089
1970s	0	244	54	200	5	9	10	1261
1980s	0	331	63	241	1	19	10	1096
1990s	1	299	71	317	5	29	12	953
2000s	1	290	52	308	2	17	8	942

^2^,

**Table 3:** The Auk

	Amensalism	Competition	Parasitism	Predation	Commensalism	Mutualism	Symbiosis	All articles
1890s	0	2	1	0	0	0	0	1663
1900s	0	10	2	0	0	0	0	1747
1910s	0	4	6	0	0	0	0	2151
1920s	0	9	24	0	0	0	0	2752
1930s	0	18	31	11	0	0	0	2781
1940s	0	43	31	57	0	0	0	2001
1950s	0	69	39	73	1	0	4	1371
1960s	0	99	68	107	1	1	0	1469
1970s	0	241	113	306	6	3	5	1984
1980s	0	447	174	455	3	13	11	2133
1990s	0	433	236	489	4	17	5	1781
2000s	0	359	228	448	4	5	5	1609

^3^,

**Table 4:** The Bryologist

	Amensalism	Competition	Parasitism	Predation	Commensalism	Mutualism	Symbiosis	All articles
1900s	0	4	0	0	0	0	0	503
1910s	0	1	4	0	0	1	3	418
1920s	0	3	1	0	0	0	0	441
1930s	0	1	0	0	0	0	5	423
1940s	0	6	1	0	0	0	1	448
1950s	0	3	1	0	0	0	6	633
1960s	0	23	4	0	0	0	23	815
1970s	0	27	2	2	0	3	41	1118
1980s	0	51	16	10	0	4	44	985
1990s	0	64	10	8	0	11	65	1045
2000s	0	74	11	6	2	3	91	923

^4^,

**Table 5:** Ecology

	Amensalism	Competition	Parasitism	Predation	Commensalism	Mutualism	Symbiosis	All articles
1920s	0	74	13	0	0	1	16	680
1930s	0	111	11	0	0	2	14	810
1940s	0	132	23	40	3	3	14	852
1950s	0	283	50	106	10	8	20	1418
1960s	0	445	64	205	5	3	18	1983
1970s	2	716	67	513	7	27	24	2111
1980s	6	1237	143	954	7	137	54	2680
1990s	3	1543	290	1213	19	227	59	3090
2000s	2	1942	376	1152	19	339	142	4252

^5^,

**Table 6:** Journal of Ecology

	Amensalism	Competition	Parasitism	Predation	Commensalism	Mutualism	Symbiosis	All articles
1920s	0	73	8	0	1	0	9	358
1930s	0	112	5	0	0	1	2	436
1940s	0	71	6	1	0	0	2	340
1950s	0	157	15	11	3	0	7	613
1960s	0	276	11	22	1	3	10	912
1970s	0	275	18	63	1	4	12	1119
1980s	0	449	21	156	5	19	29	1373
1990s	0	537	20	170	5	44	35	1353
2000s	1	786	53	248	4	95	67	1263

^6^,

**Table 7:** Journal of Mammalogy

	Amensalism	Competition	Parasitism	Predation	Commensalism	Mutualism	Symbiosis	All articles
1920s	0	18	4	1	2	0	1	584
1930s	0	16	6	13	0	0	1	805
1940s	0	31	17	69	4	0	1	753
1950s	0	75	21	140	2	0	2	1323
1960s	0	108	30	176	2	0	0	1757
1970s	0	234	20	228	0	1	0	1647
1980s	1	300	21	300	1	1	4	1622
1990s	1	369	34	399	2	10	2	1522
2000s	0	455	32	540	1	14	2	1755

^7^,

**Table 8:** Journal of Parasitology

	Amensalism	Competition	Parasitism	Predation	Commensalism	Mutualism	Symbiosis	All articles
1920s	0	2	68	0	4	0	9	531
1930s	0	1	83	0	3	1	4	883
1940s	0	4	99	5	1	0	9	984
1950s	0	25	152	3	1	3	6	1503
1960s	0	51	253	22	2	2	16	2593
1970s	2	56	286	41	8	7	55	2982
1980s	0	85	246	64	1	3	40	2371
1990s	0	112	306	69	7	7	24	2213
2000s	1	175	460	135	3	8	20	2797

^8^,

**Table 9:** New Phytologists

	Amensalism	Competition	Parasitism	Predation	Commensalism	Mutualism	Symbiosis	All articles
1910s	0	24	8	0	0	2	6	276
1920s	0	12	14	0	0	1	8	196
1930s	0	14	6	0	0	0	3	249
1940s	0	23	3	0	0	0	1	215
1950s	0	43	6	0	0	0	6	351
1960s	0	71	16	0	0	0	27	556
1970s	0	148	25	10	0	4	118	1320
1980s	1	332	52	29	1	11	282	1850
1990s	1	450	75	42	3	38	491	2170
2000s	0	666	114	78	8	104	540	3050

^9^, 10

**Table 10:** Proceedings of the Royal Society of London. Series B, Biological Sciences

	Amensalism	Competition	Parasitism	Predation	Commensalism	Mutualism	Symbiosis	All articles
1810s	0	0	0	0	0	0	0	422
1820s	0	0	0	0	0	0	0	No data
1830s	0	5	0	0	0	0	0	572
1840s	0	0	0	0	0	0	0	288
1850s	0	3	0	0	0	0	0	721
1860s	0	3	0	0	0	0	0	912
1870s	0	5	2	0	0	0	0	1066
1880s	0	10	3	0	2	2	5	1141
1890s	0	6	0	0	2	0	5	1176
1900s	0	11	11	0	0	0	3	905
1910s	0	5	5	0	1	0	6	544
1920s	0	10	1	0	0	0	3	559
1930s	0	31	20	0	0	0	4	817
1940s	0	28	4	1	0	0	4	273
1950s	0	58	18	4	1	1	14	733
1960s	0	65	17	4	2	0	3	965
1970s	0	118	23	20	0	3	48	1224
1980s	0	117	17	47	1	2	76	1153
1990s	3	749	210	402	2	67	95	2560
2000s	0	1610	483	790	6	225	115	4606

, and

**Table 11:** Science

	Amensalism	Competition	Parasitism	Predation	Commensalism	Mutualism	Symbiosis	All articles
1880s	0	322	20	0	2	1	3	7419
1890s	0	261	45	1	3	2	41	7994
1900s	0	305	98	0	10	0	31	8723
1910s	0	282	108	0	10	4	36	9111
1920s	0	745	86	1	13	5	52	11,986
1930s	0	397	142	6	7	6	39	13,750
1940s	0	399	55	12	1	2	30	13,935
1950s	0	680	106	28	9	4	50	16,945
1960s	0	1121	120	95	9	9	122	23,085
1970s	0	1498	86	311	1	23	157	21,136
1980s	1	1832	111	303	2	37	158	21,479
1990s	0	2038	88	184	2	29	124	25,417
2000s	0	2080	98	220	13	69	195	27,051

^ 11^.

## Footnotes

1 An alternative method to select search terms would involve topic modelling.

2 The use of the term ‘struggle’ in journals is not popular. Moreover, the term does not have exact interaction content as it covers various interactions and abiotic factors. While ‘cooperation’ is more popular than struggle, it is an unreliable search term: 20–80% of its hits even in biology journals do not refer to biological phenomena. ‘Mutual aid’ has become a popular term in the social organization and political arena.

3 If we presume that all seven interaction terms (four negative and three positive ones) were equally popular as research topics, we can calculate CD and ND values for the case in which no biases existed: 14% for CD values (1/7) and 57% for ND values (4/7). The above lower limits for biases are conservative, since they have higher values than these.

4 The *Journal of Ecology* focuses on the ecology of plants.

5 Some of the hits for ‘competition’ in *Science* are for non-biological phenomena.

6 I do not presume that the progress in correcting for biases involve coordinated community actions. Biases could be corrected by uncoordinated actions of individuals having different reasons/motivations for actions.

7 Data presented below confirm the conservative character of this threshold: many early percentage values for individual interaction terms in journals are less one per cent.

8 81 may appear as high, but there are over 150,000 words in Darwin (1859).

9 Kropotkin’s ideas were derived from the Russian zoologist K. F. Kessler.

10 In fact, the number of hits for competition dropped from 11 during the 1900s to 5 in the 1910s.

11 For more recent expressions of these ideas, see Bruno et al. (2003: 119), Bronstein (2009: 1160, 1161), Rodriquez-Cabal (2012: 36, 37), and Koffel et al. (2021: 3).

12 May’s (1973) results showed that predation, commensalism, and amensalism lead to stable results, whereas competition was as incapable of producing stability as mutualism. For some reason the result concerning competition has been glossed over in the literature.

13 Keller (1988) has developed a more sophisticated connection between politics/ideology and the emphasis on competition and a lack of mutualism studies in biology than Boucher et al. (1982).

14 Other ecologists have suggested that past biases have longer roots than Lewin has suggested (cf. Wiens 1977 and Strong 1980).

15 Examples being a male bias towards favouring negative interactions, positive interactions being considered boring and negative ones exciting qua topics, or capitalistic ideology biasing how researchers view nature (see Keddy 2001: 466–469).

16 There seems to be no clear motivation for the shift. The bias does not affect only ecology. Diverse biological disciplines seem to be biased (see Sect. "Percentages for interaction terms").
